# Reduction of Lead Levels in Patients Following a Long-Term, Intermittent Calcium Ethylenediaminetetraacetic Acid (EDTA)-Based Intravenous Chelation Infusions: A Prospective Experimental Cohort

**DOI:** 10.7759/cureus.11685

**Published:** 2020-11-24

**Authors:** Stephen Petteruti

**Affiliations:** 1 Physician Assistant (PA) Program, Bryant University, Smithfield, USA

**Keywords:** coronary artery disease, lead, metal intoxication, myocardial infarction

## Abstract

Chronic exposure to and the accumulation of lead has been associated with cardiovascular and all-cause morbidity and mortality. It has also been associated with accelerated declines in cognitive function and has been theorized as a contributor to essential hypertension.

This study demonstrates the capacity of intermittent infusions of calcium ethylenediaminetetraacetic acid (EDTA) to reduce the amount of lead measured by provocative urinary testing, which is considered a marker for total body lead stores. Since lead is a known toxic substance with no safe levels, reducing the amount of accumulated lead in an individual has the possibility of decreasing the risk of heart disease, dementia, and other chronic illnesses associated with lead exposure.

This study population was 15 healthy, asymptomatic patients who were evaluated for accumulated total body lead stores as part of a routine health screening. Total body lead was estimated by measuring urinary output after the patients had received intravenous (IV) calcium EDTA as a chelating agent. After establishing their baseline stored lead levels, patients received a series of intravenous chelation infusions to reduce body lead. The average number of infusions given was 14, over an average period of 24 months. After the series of chelations, there was an average reduction in the lead of 39.16% (range of 16% to 40%). All 15 subjects had a reduction in the amount of excreted lead after the series of chelation infusions.

## Introduction

Humans have been smelting lead for over 5000 years. The residue has covered the earth and, therefore, exposure is unavoidable. Lead is a pure toxin. There is no amount of exposure that is considered safe. A commonly recognized threshold for acceptable blood lead levels is less than 10 µg per deciliter. However, increased death and disease has been correlated with levels as low as 2 µg per deciliter [1]. Lead enters the human body through the lungs, across the skin, and by oral ingestion. Lead is not metabolized upon being ingested. Some of it is excreted through feces and urine. The rest is stored primarily in the bone and soft tissue. Most individuals in Western societies accumulate lead more rapidly than the rate of elimination. Since the half-life of lead in the bone and tissue can span decades, gradual and steady accumulation can occur. The lead levels of contemporary people have been shown to be similar to those of ancient Romans and are approximately 1000-fold above natural levels typical for people who do not live in industrialized societies [2].

The chronic accumulation of lead has been proposed to be a contributing factor to coronary artery disease (CAD), peripheral vascular disease (PVD), atherosclerosis, and hypertension and has been correlated with cognitive decline in humans. Also, blood lead levels have correlated with all-cause and all-cancer mortality [3-6]. Results from TACT (Trial to Assess Chelation Therapy) demonstrated a reduction in cardiovascular complications in individuals who received intravenous chelation therapy with EDTA (ethylenediaminetetraacetic acid), vitamin C, and other vitamin supplements when given intravenously to subjects who had a prior history of an MI, thus supporting the theory that lead is a risk factor for heart disease [7].

Since it appears that the entire population may be suffering from some degree of accumulated total body lead, it can become difficult to separate chronic lead exposure from its contribution to underlying disease states. Despite that limitation, there have been studies connecting elevated lead to human disease. Double-blind placebo-controlled studies on humans would obviously be unethical, thus most of the correlations with the disease have come from observational studies. A study published in 2013 concluded, “blood lead concentration correlates with all-cause, all cancer, and lung cancer mortality in adults” [5]. Another study published in 2018 calculated that lead contributed to over 400,000 deaths annually from cardiovascular and ischemic heart disease in the US [8]. These and other studies have caused us to reconsider the generally accepted notion of what is a safe blood level. In fact, most experts feel that there is no such thing as a safe level of lead since it is a pure toxin and the adverse effects seem to correlate with the level of accumulation.

The cause-and-effect of neurocognitive insult and clinical consequence can sometimes be delayed by decades, thereby making it difficult to appreciate factors contributing to cognitive decline [9]. A paper published in 2004 from the VA Normative Aging Study noted a correlation between the amount of lead stored in bone and a decline in cognitive function. It should be noted that the individuals in the study did not have occupational exposures or known risk factors for lead accumulation [10]. In addition to cardiovascular and cognitive consequences, chronic lead exposure may be associated with immune function alterations that may increase the risk of atopic autoimmune diseases, as well as weakening host immune defense and possibly contributing to increased cancer risk [11-12].

The current study was undertaken to determine if less frequent intervals of chelation could significantly lower lead levels. Calcium EDTA was chosen as a chelating agent as opposed to disodium EDTA that was used in the TACT trial. Calcium EDTA can be administered much more quickly, typically over one hour versus three hours needed for disodium. This contributes to making the treatment more cost-effective. It is also regarded as a safer alternative to Na EDTA. The objective was to have subjects receive an infusion every month. This was done to make the treatments more affordable and less of an imposition to patients.

## Materials and methods

Methodology

This was a prospective experimental cohort study made up of 15 clients who voluntarily enrolled for chelation treatment. Clients in this cohort had their first heavy-metal test obtain between April 2015 and August 2018. In order to be considered for treatment, individuals had to be free of significant renal or cardiovascular disease. They also had to have a history that was negative for known high-level exposure to lead or other toxic heavy metals. The median age of the subjects was 64 years. They performed an average of 14 chelations (range 6-30) over an average of 24 months. Six of the 15 were men; the remaining nine were women. All of the subjects were Caucasian. Two of the subjects, both males, had a prior history of coronary artery disease with bypass grafting. None of the subjects were diabetic.

The setting was a private independent vitamin infusion center (“Intellectual Medicine 120”). All of the clients signed written informed consent regarding the risks and benefits of chelation therapy, as well as the limitations regarding its clinical expectations. They all understood that the objective was to reduce the total body burden of lead and that they were not being treated for "lead toxicity." The clients presented from the surrounding community on a voluntary basis, seeking infusions for general health support. None of them had occupational exposure to heavy metals. None of them had a history of known lead toxicity or prior exposures. 

Materials

All of the subjects received a provocative heavy-metal test. Clients first received 3 g of calcium EDTA, then they collected their urine for six hours and sent a specimen of the collected urine for analysis. The urine specimen was processed by Doctors Data, Saint Charles, Illinois. The statistical data were analyzed, and the t-test was performed by an independent statistical analyst (Kory Keough BS; Research, Assessment, and Outcome Analyst at Bryant University, Smithfield, RI). 

For this study, it was decided to forgo a non-provoked urine heavy-metal test. The non-provocation test is used to assess recent exposure to heavy metals, typically over the prior two weeks. Although this approach has merit to rule out factors that could skew the provocative study, given the fact that the subjects had no historical exposures, it was decided that the pre-test probability of significant findings was low and that the cost/benefit analysis did not favor adding this test to the patient's treatment regimen.

All of the subjects had an initial health screening, which included a physical exam and blood work, including calcium and creatinine levels. None of the subjects had renal insufficiency, and they all had creatinine levels less than 1.5.

After signing informed consent, subjects received infusions of the following chelation mix:

Calcium EDTA - dose calculated individually* (formula listed in the Appendix).

Vitamin C - 7500 mg

Magnesium - 1500 mg

B12 - 1000 mg

B complex - 1 ml

Glutathione - dose pushed separately at 400 mg

No other therapies were required in conjunction with chelation therapy. There were no pre-specified oral vitamins or supplements that were required. None of the subjects were restrained from pursuing other healthcare support as they saw fit. None of them were using oral chelating agents.

The decision regarding when to repeat heavy-metal testing was not at a predetermined interval. The repeat heavy-metal test was performed after at least 10 chelations or after at least 10 months since the start of the first chelation.

## Results

**Figure 1:**
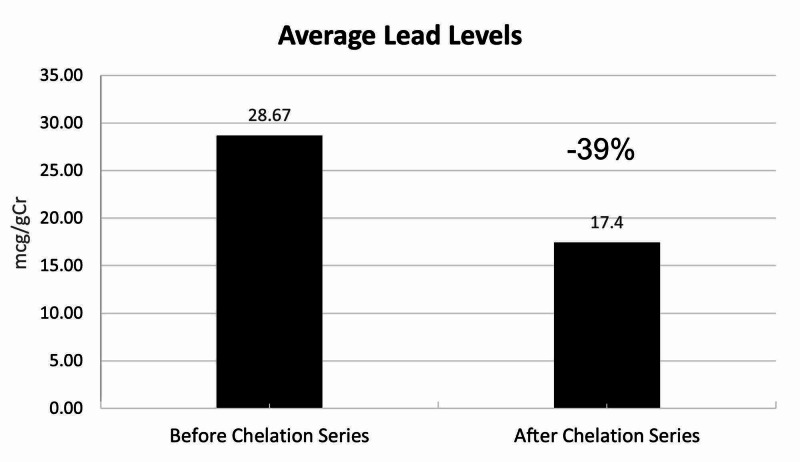
Average Lead Levels The bar graph represents the average lead levels of all 15 subjects. The bar on the left represents the average lead levels prior to the chelation series. The bar on the right represents the average lead levels after the chelation series was completed (P score of 0.00004).

The median age of the subjects was 64 years. All of the patients were Caucasian. There were six men and nine women in the study. Two of the patients had prior cardiac bypass surgery. None of the patients were diabetic. Patients received an average of 14 Infusions (range 6 to 30). The infusions were delivered over an average time period of 24.2 months (range 6 to 40). The provoked levels of lead prior to the series of chelations was an average of 28.6 mcg/gCr (range 12 to 64). The average level of provoked lead after the series was 17.4 mcg/gCr (range 4 to 54) (Table 1) This difference calculated to a P score of 0.00004 (Figure 1).

**Table 1:** Patient Information Patients (n = 15) age, sex (male (M) or female (F)), number of chelation infusions, the time span in months for infusions, year and month of the first test, provoked lead level prior to infusion series, year and month of the second test, provoked lead level after infusion series, percent of change, and start versus end creatinine (Cr).

Patient	Gender	Age	Number of Chelation Infusions	Time Span in Months for Infusions	First Heavy Metal Test	Lead Level Before Chelation Series (mcg/gCr)	Second Heavy Metal Test	Lead Level After Chelation Series (mcg/gCr)	Change (Before & After)	Percent of Change	Start Cr	End Cr
1	M	60	20	37	6/16	21	7/19	16	-5	-24	0.95	0.96
2	F	67	23	21	3/18	41	12/19	20	-21	-51	0.95	Unavailable
3	M	67	10	12	8/18	20	8/19	12	-8	-40	0.95	Unavailable
4	F	70	9	19	1/18	30	10/19	21	-9	-30	0.95	0.8
5	F	68	10	6	6/18	28	12/18	11	-17	-61	0.95	0
6	M	75	13	29	7/17	16	12/19	11	-5	-31	0.9	0.9
7	M	78	13	8	8/17	64	4/18	54	-10	-16	0.84	0.86
8	F	57	9	21	3/17	31	12/18	21	-10	-32	0.95	0.91
9	F	64	10	26	7/16	12	9/18	4	-8	-67	0.7	0.73
10	F	74	18	25	12/17	55	1/20	21	-34	-62	0.81	0.6
11	F	64	10	10	2/18	14	12/18	6	-7.6	-57	0.62	0.51
12	F	61	19	36	9/15	31	5/18	24	-7	-23	0.82	0.83
13	F	33	6	40	11/15	16	3/19	7	-9	-56	0.78	0.75
14	M	46	11	39	4/15	37	9/18	22	-15	-40.5	1	0.87
15	M	55	30	34	9/15	14	7/18	11	-3	-21.4	1.2	1.09
Average		62.6	14.07	24.2		28.67		17.4	-11.24	-41.99	0.89	0.75

The average creatinine level prior to the infusions was 0.89. After the infusions, the average was 0.75. None of the patients had an increase in creatinine over the course of the infusions. Two of the patients did not have a post-infusion creatinine level recorded. These results are in keeping with other studies on chelation therapy that have not found any adverse effect on kidney function [7,13-14].

None of the subjects reported side effects during or after the chelation therapy.

## Discussion

There is a universal consensus that lead is a potent, absolute toxin. Exposure to any amount is considered harmful. There is also a consensus that the amount of lead in the environment has increased since the dawn of the industrial revolution. The environmental lead includes not only that which is in the water and in the air but that which is absorbed into the foods that we eat. In summary, lead is impossible to avoid. The question is not whether or not we have accumulated lead in our bodies but how much.

Given that exposure to lead is ubiquitous and that lead is a pure toxin, it presents a universal risk factor for multiple chronic diseases. Since it is a risk factor that is distributed throughout developed nations, it becomes difficult to discern to what extent baseline lead exposure is affecting the health of individuals. Therefore, isolating lead as a health risk factor becomes nearly impossible to do in a manner similar to traditional cohort studies. Unlike other risk factors, such as cigarette exposure in individuals with lung cancer, lead exposure is universal. There is no population-based cohort that can be followed in order to determine the difference between quartiles of lead accumulation and corresponding disease states. In that sense, the amount of lead that humanity is exposed to represents a foundational risk factor that may increase the risk of all disease states that it contributes to. In order to determine the impact of reducing total body lead on health outcomes, a prospective cohort study should be designed that would be sufficiently powered, and span a long enough time, to determine the correlation between lead reduction by intravenous chelation and outcomes such as heart disease and dementia. Other studies have demonstrated reduced lead levels by chelation but with the use of more frequent and greater numbers of infusions [13-14]. In order for chelation therapy to be practical for a large segment of the population, it must prove to be affordable in terms of cost per infusion and in regard to the frequency of infusions required. This is especially true because chelation therapy, when used for health preservation rather than disease intervention, is an out-of-pocket expense to clients. 

For the purpose of our patient cohort, we decided to use calcium EDTA rather than disodium EDTA. We decided to use calcium rather than disodium EDTA for several reasons. First, it can be administered over a shorter period of time, reducing infusion from three hours, down to one hour. This reduces the cost of the infusion and makes it less of a time burden. Secondly, calcium EDTA has a long-established history of safety with less risk of disturbed serum calcium levels. Finally, based upon its mechanism of action, calcium EDTA is expected to have the same value with regard to cardiovascular outcomes as disodium EDTA.

Our cohort had 15 out of 15 individuals with a reduction in their lead levels. This is a higher percentage of individuals with reduced lead levels when compared to other studies where disodium EDTA was used. While this could be by chance, it is possible that the calcium EDTA is a more effective chelator or that the addition of glutathione in our protocol enhanced lead reduction. Glutathione was added to the infusion because of its antioxidant benefit and its potential to mitigate lead-induced oxidative damage.

Limitations

This was a small study with no control group. Each patient acted as their own control. The lack of predetermined intervals for the chelation therapy and the wide range of the total number of infusions performed are also weaknesses of this study. Another limitation of the study is the lack of ethnic diversity amongst the patients. The P score for this study supported the null hypothesis, indicating a high likelihood that the outcomes were the result of chelation therapy. However, the small number of cases in this cohort represents a limitation with regard to interpreting the power of the outcome. Despite these limitations, this preliminary study demonstrates the potential for intermittent infusions of calcium EDTA to reduce total body lead stores.

## Conclusions

This study demonstrates a practical approach toward the reduction of chronic accumulated lead. It provides evidence that monthly (or less often) infusions of calcium EDTA along with other vitamins can reduce total body lead levels. Although it remains unknown whether or not such an approach would have an impact on disease outcomes, accumulating evidence connecting chronic lead to heart disease and cognitive decline supports the possibility that regular chelation therapy may be beneficial for heart and brain health, as well as possibly reduce the risk of cancer and other diseases. In the face of a known toxin, it is logical to expect that reducing the toxin will diminish health risk. By reducing the frequency of the chelations and extending them over time, the cost of receiving treatment is significantly reduced, thus making it a rational consideration for disease prevention and health maintenance.

## Appendices

Formula for calculating Ca-EDTA dose

EDTA dose mg = 50 mg x LBW x CrCl/100

(LBW = Lean Body Weight. For men = 50 kg + 2.3 kg for every 1” over 5’. For women = 45 kg + 2.3 kg for every 1” over 5’)
